# HPSE-mediated proinflammatory signaling contributes to neurobehavioral deficits following intranasal HSV-1 infection

**DOI:** 10.1128/mbio.03765-24

**Published:** 2025-02-27

**Authors:** Hemant Borase, Chandrashekhar D. Patil, Tibor Valyi-Nagy, Deepak Shukla

**Affiliations:** 1 Department of Ophthalmology and Visual Sciences, University of Illinois Chicago 12247 , Chicago, Illinois, USA; 2 Department of Pathology, Neuropathology Service, University of Illinois Chicago, Chicago, Illinois, USA; 3 Department of Microbiology and Immunology, University of Illinois Chicago, Chicago, Illinois, USA; Huazhong Agricultural University, Wuhan, Hubei, China

**Keywords:** heparanase, herpesviruses, inflammation, toll-like receptors, caspases, microglia, Behavior

## Abstract

Herpes simplex virus-1 (HSV-1) is a neurotropic virus that can infect the brain, and an uncontrolled infection can lead to a range of diseases, including chronic nerve pain, encephalitis, and neurobehavioral abnormalities. These outcomes are often severe and have lasting consequences, highlighting the need to identify host factors that contribute to disease severity. In this study, we report that intranasal HSV-1 infection in murine model, which promotes viral dissemination into the brain, implicates the host protein heparanase (HPSE) as a key mediator of neuroinflammation. Specifically, we observed that the HPSE activity during HSV-1 infection in naïve animals promotes the upregulation of proinflammatory cytokines, enhances microglial activity in the brain, and contributes to cognitive impairment, anxiety, and motor coordination deficits. Such effects are significantly less detectable in heparanase deficient (*Hpse−/*−) mice. Additionally, we found that moderate activation of toll-like receptors (TLRs), particularly in *Hpse+/+* mice, may contribute to the activation of the inflammasome pathway. This, in turn, leads to the activation of caspase-1 (*Casp1*) and caspase-3 (*Casp3*), which may play a role in nerve function loss. Our findings position HPSE as a potential therapeutic target for mitigating virus-induced neuroinflammation and neurobehavioral defects.

**IMPORTANCE:**

Herpes simplex virus-1 (HSV-1) infection in the brain can lead to severe and often permanent neurological consequences. Host factors influence disease outcomes in response to infection, and understanding these factors is crucial for developing effective therapies. This study identifies the host protein HPSE as a key mediator of neuroinflammation in response to HSV-1 infection. We demonstrate that the HPSE activity drives proinflammatory cytokine expression and microglial activation and promotes a signaling cascade involving toll-like receptors and caspase activation, potentially intensifying neuroinflammatory responses. These findings implicate HPSE as an important player in HSV-1 pathogenesis in the central nervous system and suggest that targeting HPSE could provide a novel therapeutic strategy to mitigate virus-induced neuroinflammation and neurobehavioral disturbance.

## INTRODUCTION

Neuroinflammation is a hallmark of many viral infections affecting the central nervous system (CNS). It is a major driver of neurological disorders, including Alzheimer’s disease (AD), Parkinson’s disease (PD), amyotrophic lateral sclerosis (ALS), and Huntington’s disease (1, 2). It is predicted that by 2050, approximately 141 million cases of AD will be reported worldwide (3). Current research on CNS inflammation has shown that the resulting production of inflammatory cytokines and reactive oxygen species plays a significant role in neurodegeneration, suggesting potential therapeutic targets to address neuroinflammation in neurodegenerative diseases (4).

Herpes simplex virus-1 (HSV-1) is a highly prevalent pathogen that substantially impacts global health, contributing to pathological conditions, such as cold sores, genital herpes, and herpetic stromal keratitis. Its ability to establish latency in the trigeminal ganglion (TG) and reactivate in response to stress complicates eradication efforts (5). While HSV-1 is a known cause of viral encephalitis, leading to blood-brain barrier breakdown and leukocyte infiltration, the virus’ spread to the CNS via the olfactory route can also be rapid and particularly destructive. Although intranasal infection is rare in humans, this route more directly disseminates the virus to the brain, causing acute and chronic damage that could result in long-term debilitating outcomes, including neuronal loss, neurodegeneration, and behavioral deficits. Similar symptoms are reported in individuals recovering after more serious consequences of HSV-1 encephalitis. HSV-1 infection and reactivated virus spread in the brain can also lead to severe neuroinflammation and immune response, exacerbating neuronal injury and cognitive impairments (3, 6).

In cases of both animal models and humans, HSV-1 has been detected in brain tissue postmortem and cerebrospinal fluid, even without clinical symptoms of encephalitis (7, 8). This suggests the virus can establish long-term persistence within the CNS, and immunological response to the viral presence may trigger chronic neuroinflammatory events like those seen in chronic neurodegenerative diseases. Systemic inflammation and neuroinflammatory pathways activated by HSV-1 have been implicated in neuronal dysfunction, resulting in cognitive deficits, anxiety, motor impairments, and other neurobehavioral abnormalities (9, 10). Importantly, neuroinflammation often involves hyperglial cell response, a common factor in neurobehavioral disorders, highlighting the need to better understand viral-mediated neuroinflammatory mechanisms (11).

Heparanase (HPSE) is a tissue-remodeling enzyme or an endoglycosidase that degrades heparan sulfate in the extracellular matrix (12–15). It also exerts pro-inflammatory effects in various viral infections and cancers (16). HPSE enhances viral pathogenesis by inducing chemokines and cytokines, thereby promoting inflammation (17–19). Heparan sulfate, the substrate for HPSE, is known to facilitate HSV-1 entry inside the cell and reported for its role in inflammation (20, 21).

In the context of HSV-1 infection, HPSE has been shown to facilitate viral release and propagation in corneal epithelium, and several host molecules, such as cyclic-AMP-responsive element-binding protein 3 (CREB3) and protein kinase-B (AKT), were reported to interact with HPSE (5, 21–25). HPSE’s essential role in homeostasis, metastasis, angiogenesis, autophagy, and DNA damage was studied extensively, yet its role in brain infection and neuroinflammation remains underexplored (25–27).

We hypothesize that HPSE contributes to neuroinflammatory damage in HSV-1 infection of the CNS, leading to neuronal loss and neurobehavioral impairments. To test our hypothesis, we utilized wild-type (*Hpse+/+*) and heparanase deficient (*Hpse−/*−) mice. Using intranasal HSV-1 infection to simulate more direct viral dissemination to the brain, we investigated the key markers of HPSE-driven neuroinflammation, regulation, and its effects on cognitive function, anxiety, and motor coordination. By demonstrating HPSE’s role in neuroinflammation and its downstream impact on neurobehavioral function, we provide evidence supporting the therapeutic potential of HPSE inhibition to mitigate HSV-1-induced CNS damage (28). Our findings have implications for understanding HSV-1’s role in brain inflammation and neurodegeneration and suggest that targeting HPSE may be beneficial in managing the long-term neurological outcomes of viral infections.

## RESULTS

### Heparanase exacerbates HSV-1 infection, brain tissue remodeling, and mortality after intranasal virus administration

**Fig 1:**
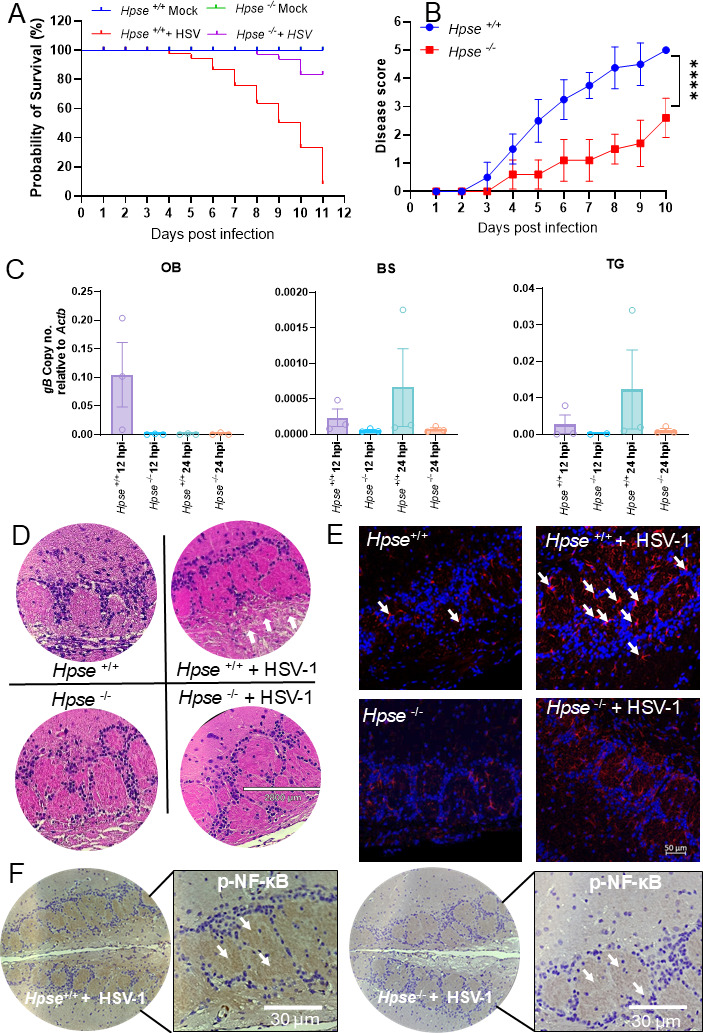
Heparanase intensifies inflammation, morbidity, and mortality after HSV-1 infection in *Hpse+/+* mice. (**A**) Survival of *Hpse+/+* and *Hpse−/*− mice after intranasal infection at different days post-infection. (**B**) Disease score of *Hpse+/+* and *Hpse−/*− mice based on identified disease parameters after HSV-1 infection. (**C**) Viral m-RNA transcripts from different regions of the brain [olfactory bulb (OB), brain stem (BS), and trigeminal ganglion (TG)] after giving HSV-1 infection at 12 and 24 hpi. (**D**) H&E staining of OL of mock and HSV-1 infected *Hpse+/+* and *Hpse−/*− mice (arrows indicate tissue necrosis in *Hpse+/+* mice after infection). (**E**) Immunofluorescence staining for IBA1 (microglia) in OL after chronic HSV-1 infection (arrows indicate microglial cells). (**F**) Immunohistochemistry of pNF-κB in OL of *Hpse+/+* and *Hpse−/*− mice after chronic HSV-1 infection (arrows indicate p-NFKB positive area) (*n* = 3 mice per group). One- and two-way ANOVAs with multiple comparisons were used for statistical analysis. *P < 0.05; **P < 0.01; ***P < 0.001, and ****P < 0.0001.

Our previous work has highlighted the role of HPSE in driving pathological responses on the ocular surface after HSV-1 infection (16, 18, 28, 29). To extend this understanding to neuroinflammation and host survival, we hypothesized that intranasal administration of HSV-1 would exacerbate disease progression, and that HPSE would be a key contributor to this enhanced pathology. Using *Hpse+/+* and *Hpse−/*− mouse models with intranasal HSV-1 infection, we observed significantly higher infection rates, morbidity, and mortality in *Hpse+/+* mice compared to *Hpse−/*− mice, suggesting a critical role of HPSE in HSV-1-related pathology (Fig. 1A). *Hpse+/+* mice exhibited an earlier onset of mortality, and clinical scores indicated more rapid disease progression in *Hpse+/+* than in *Hpse−/*− mice (Fig. 1B). Viral glycoprotein mRNA transcripts were notably upregulated in brain regions, such as the olfactory bulb (OL), brainstem (BS), and trigeminal ganglion (TG) in *Hpse+/+* mice, reflecting increased viral presence compared to *Hpse−/*− mice (Fig. 1C). Additionally, *ex vivo* reactivation of HSV-1 on days 5 and 10 post-infection revealed greater reactivation in the TG of *Hpse*+/+ mice likely due to heightened neuroinflammation, whereas HSV reactivation was limited in *Hpse−/*− mice (Fig. S1A). To investigate long-term impacts, *Hpse+/+* and *Hpse−/*− mice were infected with a sub-lethal dose of HSV-1. At 6 months post-infection, hematoxylin and eosin (H&E) staining revealed structural alterations of pathological significance in the OL of *Hpse+/+* mice. More signs of dead cells and inflammation were observed in *Hpse+/+* mice infected with HSV-1 (white arrows Fig. 1D). In contrast, *Hpse−/*− mice OL displayed fewer morphological changes and fewer indications of dead cells and inflammation (Fig. 1D). *Hpse+/+* mice exhibited elevated microglial populations in the OL, while microglial numbers were relatively lower in *Hpse−/*− mice brains (Fig. 1E; Fig. S1B). Lastly, pNF-κB, a known marker of inflammation (21), was higher in *Hpse+/+* OL compared to OL of the *Hpse−/*− animal (Fig. 1F). These findings demonstrate that HPSE enhances neuroinflammatory responses and pathognomonic remodeling of the brain tissue architecture, intensifying the impact of HSV-1 on the host.

### Heparanase promotes HSV-1-induced neuroinflammation

**Fig 2:**
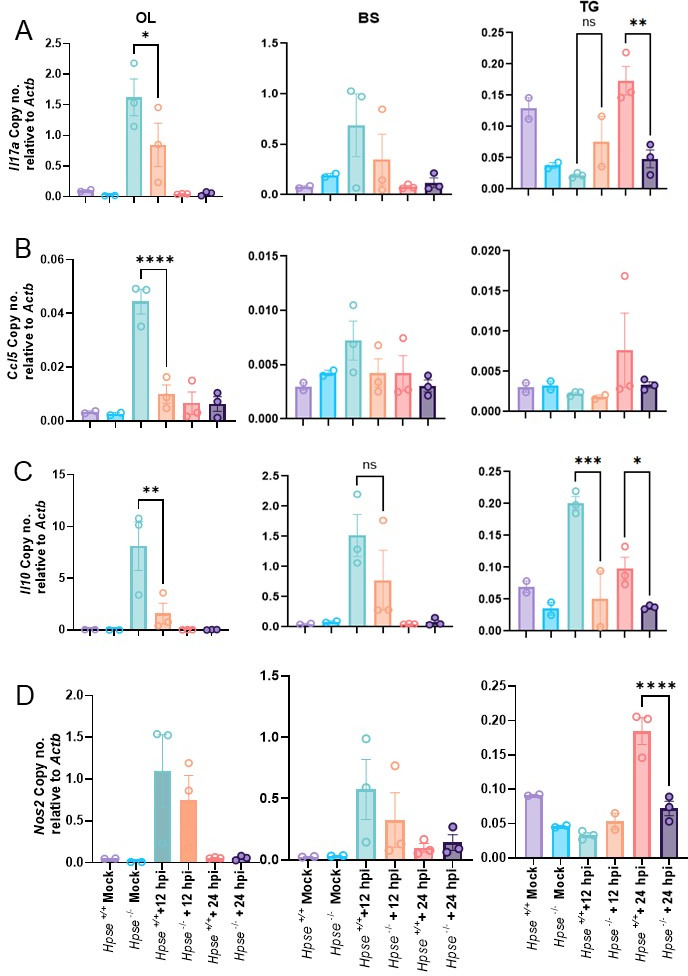
HPSE increases cytokines and *Nos2* expression after HSV-1 infection. Bar graph showing the m-RNA transcripts from OL, BS, and TG of *Hpse+/+* and *Hpse−/*− mock and HSV-1 infection after 12 and 24 hpi (**A**) *Il17a*, (**B**) *Ccl5*, (**C**) *Il10*, and (**D**) *Nos2*. (*n* = 2 mice per mock group and 3 mice per HSV-1-infected group.) Ordinary one-way ANOVA with multiple comparison was used for statistical analysis. *P < 0.05; **P < 0.01; ***P < 0.001, and ****P < 0.0001.

To further understand how HPSE amplifies neuroinflammation and HSV-1 pathogenesis, we investigated its influence on other host inflammatory factors. Previous studies from our group demonstrated HPSE’s role in enhancing inflammatory responses in corneal cells (18, 21). Given that we used an intranasal route for HSV-1 infection, we hypothesized that HPSE would similarly intensify inflammatory processes within the brain. Consistent with this hypothesis, we observed significantly elevated mRNA levels of the key pro-inflammatory cytokine, *Il17a*, and chemokine, *Ccl5*, in the OL and TG of *Hpse+/+* mice compared to *Hpse−/*− mice (Fig. 2A and B). The significantly increased expression of the key anti-inflammatory cytokine, *Il10*, in OL and TG of *Hpse+/+* mice also provides additional evidence of an inflammatory environment and tissue damage (Fig. 2C). Moreover, nitric oxide synthase 2, inducible (*Nos2*), an enzyme that produces reactive nitrogen species in response to inflammatory stimuli, was also significantly upregulated in *Hpse+/+* mice. This upregulation likely exacerbates the inflammation induced by cytokine release, further intensifying tissue damage and contributing to prolonged neuroinflammatory conditions (Fig. 2D). Histological analysis supported these findings, revealing substantial structural alterations in *Hpse+/+* brains that were minimized in *Hpse−/*− mice after HSV-1 infection. These data underscore HPSE-driven inflammation as a critical factor driving severe HSV-1-induced neuroinflammation and associated pathology.

### Tissue-specific variation in TLR expression after HSV-1 infection

**Fig 3:**
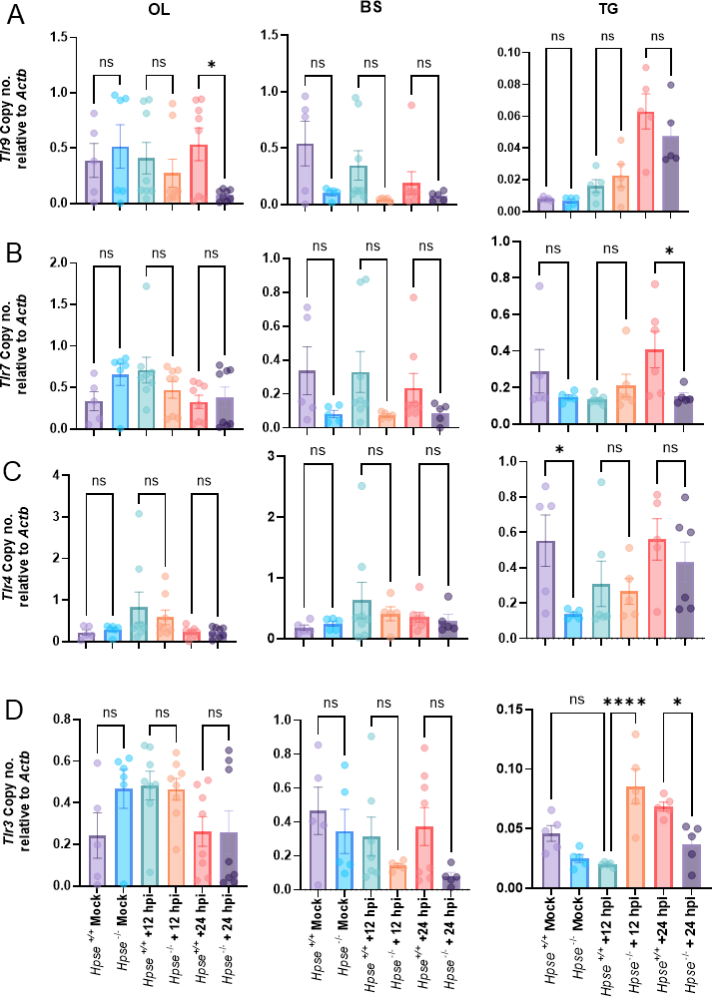
Tissue-specific variation in TLR expression after HSV-1 infection. Bar graph showing the m-RNA transcripts from OL, BS, and TG of *Hpse+/+* and *Hpse−/*− mock and HSV-1 infection after 12 and 24 hpi. (**A**) *Tlr9*, (**B**) *Tlr7*, (**C**) *Tlr4*, and (**D**) *Tlr3*. (*n* = 5). Ordinary one-way ANOVA with multiple comparison was used for statistical analysis. *P < 0.05; **P < 0.01; ***P < 0.001, and ****P < 0.0001.

After identifying elevated inflammatory markers in the brains of *Hpse+/+* mice following HSV infection, we examined toll-like receptors (TLRs), which are key players in recognizing viral components and initiating immune responses. Previous studies have shown that TLR-mediated signaling cascades can amplify inflammation and oxidative stress (30). Therefore, we measured the expression of various TLRs in both *Hpse+/+* and *Hpse−/*− mice, as these receptors have been previously linked to HSV-1 infection (Fig. 3).

Overall, we observed tissue-specific, differential responses to multiple TLRs in *Hpse+/+* and *Hpse−/*− mice at different time points. In the olfactory lobes (OL), there were no significant differences in the expression of *Tlr3*, *Tlr4*, or *Tlr7*, although *Tlr9* expression moderately increased at 24 h post-infection (hpi) in *Hpse+/+* mice (Fig. 3A). The OL is a primary site of neuronal HSV-1 infection via the nasal route, suggesting that HPSE may potentiate TLR activity in response to HSV-1 infection, particularly through this route.

No significant differences in TLR expression were observed in the brainstem of either *Hpse+/+* or *Hpse−/*− mice at 12 or 24 hpi (Fig. 3A through D). In the trigeminal ganglion (TG) of *Hpse+/+* mice, we noted a moderate increase in *Tlr7* expression at 24 hpi (Fig. 3B), as well as higher baseline levels of *Tlr4* expression (Fig. 3C). Interestingly, a significant increase in *Tlr3* expression was observed in *Hpse−/*− mice at 12 hpi (Fig. 3D). To conclude, the moderate increase in certain TLRs in *Hpse+/+* mice may be associated with a secondary response to HSV-1 infection. These findings suggest that HPSE likely promotes TLR activation, which detects viral RNA and triggers immune responses, potentially contributing to sustained inflammation and tissue injury.

### Inflammasome activation markers are upregulated in the nervous system tissues of WT mice post-HSV-1 infection

**Fig 4:**
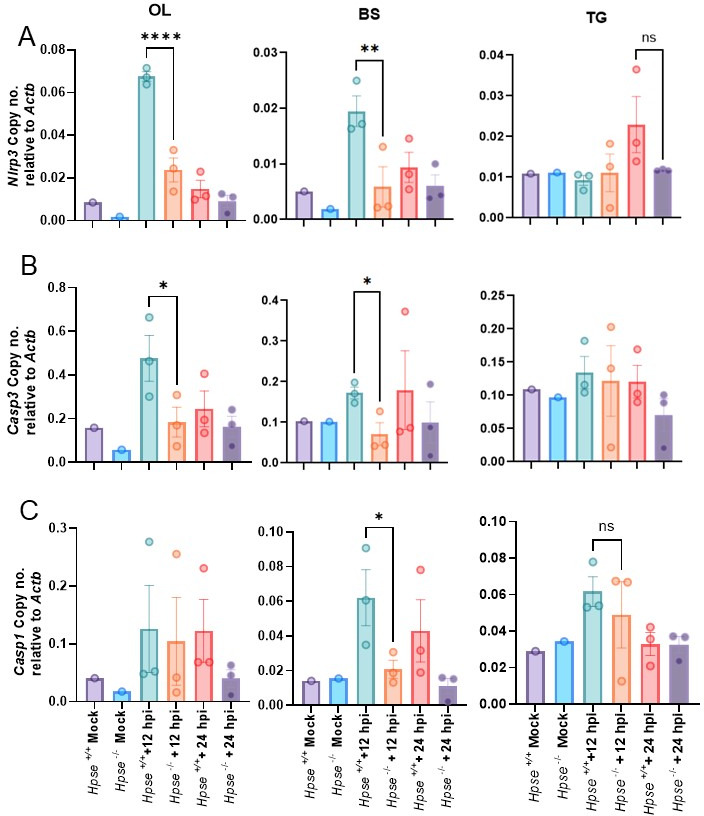
HPSE increases inflammasome formation and cell death after HSV-1 infection. Bar graph showing the m-RNA transcripts from OL, BS, and TG of *Hpse+/+* and *Hpse−/*− mock and HSV-1 infection after 12 and 24 hpi (**A**) *Nlrp3*, (**B**) *Casp3*, and (**C**) *Casp1* (*n* = 2 mice per mock group and 3 mice per HSV-1 infected group). Ordinary one-way ANOVA with multiple comparison was used for statistical analysis. *P < 0.05; **P < 0.01; ***P < 0.001, and ****P < 0.0001.

Inflammasome formation driven by host detection of damage-associated molecular patterns like TLRs plays a significant role in propagating inflammatory responses (31). Given the overall moderate TLRs upregulation observed in *Hpse+/+* animals, we investigated downstream inflammasome components. Notably, we found heightened mRNA levels of *Nlrp3*, *Casp1*, and *Casp3* in the OL and BS of *Hpse+/+* mice, with *Casp1* expression particularly elevated in the BS (Fig. 4A through C). Inflammasome activation leads to the maturation of proinflammatory cytokines, such as *IL-1β*, that further intensify neuroinflammation and exacerbate neuronal damage. This pattern of inflammasome activation highlights a cascade in which HPSE-driven TLR signaling promotes *Nlrp3* inflammasome activation, amplifying the neuroinflammatory response in HSV-1 infected *Hpse+/+* mice brains.

### HPSE triggers GSDMD-mediated pyroptosis in HSV-1-infected cells

**Fig 5:**
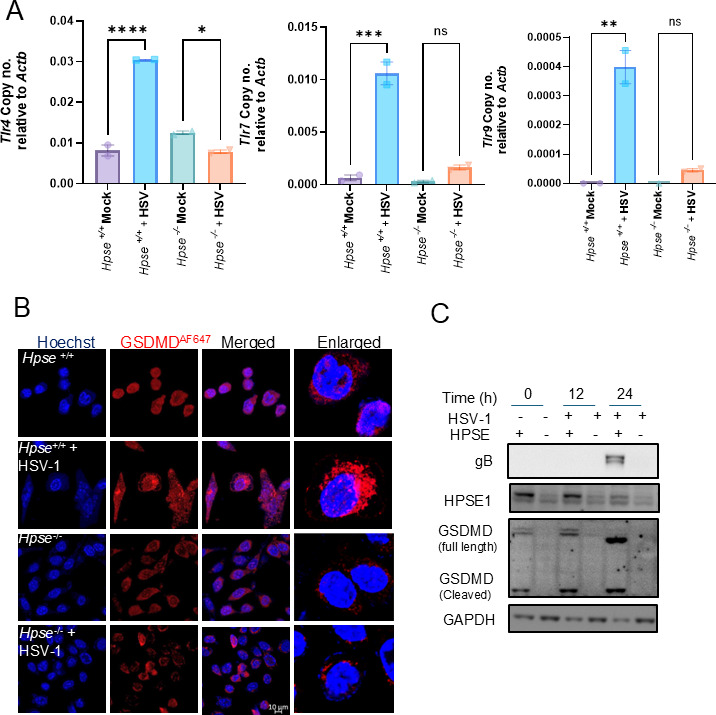
HSV-1 infection increases TLR activation and pyroptosis in MEFs cells. (**A**) m-RNA transcript from *Hpse+/+* and *Hpse−/*− MEFs after infection of HSV-1-KOS WT virus at 5 MOI for 12 h (*n* = 2 each group). (**B**) Confocal imaging for gasdermin D from *Hpse+/+* and *Hpse−/*− MEFs after infection of HSV-1-KOS WT virus at 5 MOI for 12 h. (**C**) Western blot for gasdermin D (+ = *Hpse + /+* MEFs and − = *Hpse−/*− MEFs) from *Hpse+/+* and *Hpse−/*− MEFs after infection of HSV-1-KOS WT virus at 0.1 MOI for 12 and 24 h. Ordinary one-way ANOVA with multiple comparison was used for statistical analysis. *P < 0.05; **P < 0.01; ***P < 0.001, and ****P < 0.0001.

Based on the earlier results from brain and TG, we were interested to know if the response is specific to neural cells or represents a more generalizable effect; hence, we examined *Tlr4*, *Tlr7*, and *Tlr9* expressions in *Hpse+/+* and *Hpse−/*− mouse embryonic fibroblasts (MEFs) as a non-neural cellular model. By comparing TLR responses in these cell lines, we aimed to confirm HPSE’s role in modulating TLR-mediated inflammatory signaling following HSV-1 infection. Upon HSV-1 infection, *Hpse+/+* MEFs exhibited significantly elevated expression of *Tlr4*, *Tlr7*, and *Tlr9* compared to mock-infected cells, indicating an upregulated innate immune response in the presence of HPSE (Fig. 5A). The results from MEFs partially mirrored the observations in *Hpse*+/+ OL and TG, where TLRs were particularly responsive to viral RNA, thereby enhancing the inflammatory response. In contrast, *Hpse−/*− MEFs showed minimal to no upregulation of *Tlr4*, *Tlr7*, and *Tlr9* upon HSV-1 exposure. However, further research is needed to better understand the role of TLRs in infection, particularly in *Hpse+/+* MEFs and animal models. In MEFs, the HPSE-dependent increase in *Tlr4*, *Tlr7*, and *Tlr9* expressions likely amplifies downstream inflammatory responses, supporting the hypothesis that HPSE plays a central role in heightening inflammatory signaling cascades in HSV-1-infected cells. We next examined the downstream effects on inflammasome signaling pathways. HPSE mediated inflammatory cascades found to promote inflammasome assembly and pyroptotic cell death, marked by gasdermin D (GSDMD) cleavage. Cleaved GSDMD creates pores in the cell membrane, leading to cell lysis and the release of inflammatory cytokines, which further amplify local inflammation. Fluorescent staining for GSDMD in HSV-1-infected *Hpse+/+* MEFs revealed a marked increase in cleaved GSDMD, indicating active pyroptosis in infected cells (Fig. 5B). In contrast, cleaved GSDMD was minimally detected in both mock-*Hpse+/+* MEFs and in mock- and HSV-1-infected *Hpse−/*− MEFs, suggesting that HPSE is required for inflammasome activation and GSDMD-mediated pyroptosis in response to infection. Protein analysis further confirmed an increase in cleaved GSDMD in HSV-1-infected *Hpse+/+* MEFs, with little to no cleavage observed in *HPSE−/*− MEFs post-infection, underscoring HPSE’s role in inflammasome-driven pathology (Fig. 5C). Additionally, HSV-1 glycoprotein B (gB) and HPSE-1 were elevated in infected *Hpse+/+* samples, whereas gB levels were significantly lower in *Hpse−/*− MEFs post HSV-1 infection (Fig. 5C). This finding aligns with prior evidence suggesting that HPSE enhances viral persistence and exacerbates inflammation and tissue damage through prolonged viral–host interactions.

### Neurobehavioral abnormalities are reduced in HPSE−/− mice following persistent HSV-1 infection

**Fig 6:**
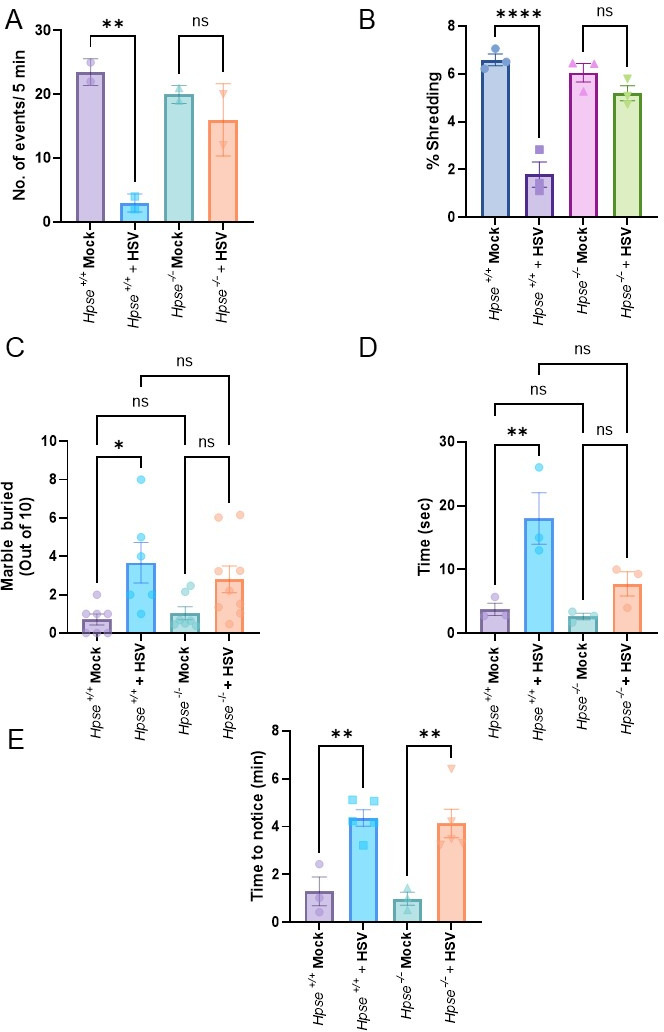
Presence of HPSE leads to behavioral abnormalities after chronic HSV-1 infection. (**A**) Novel object recognition test. (**B**) Nestlet dhredding test. (**C**) Marble burying test. (**D**) Ledge test. (**E**) Tape removal test. Ordinary one-way ANOVA with multiple comparison was used for statistical analysis. *P < 0.05; **P < 0.01; ***P < 0.001, and ****P < 0.0001.

Neuronal damage from HSV-1 infection has been associated with neurobehavioral abnormalities, including deficits in memory, anxiety, and motor coordination (32). To assess the impact of HPSE on these neurobehavioral outcomes following intranasal HSV-1 inoculation-promoting brain infection, we conducted a series of cognitive and behavioral tests on chronically infected *Hpse+/+* and *Hpse−/*− mice (Fig. S2). In the novel object recognition (NOR) test, which assesses cognitive function and memory, *Hpse−/*− mice spent significantly more time exploring novel objects compared to *Hpse+/+* mice, which displayed a lower preference for the novel object after long-term HSV-1 infection. Poor performance in the NOR test is associated with deficits in recognition memory, likely reflecting damage to hippocampal neurons. These results suggest that *Hpse−/*− mice retain better cognitive function in the face of persistent infection potentially due to reduced neuroinflammatory damage to memory-supporting neuronal circuits (Fig. 6A). In the nestlet shredding test, which assesses anxiety-related behavior through engagement in nest-building, HSV-1- infected *Hpse+/+* mice displayed a significant reduction in shredding, indicative of increased stress and anxiety. Conversely, *Hpse−/*− mice showed no detectable reduction in nestlet shredding post-infection, maintaining normal engagement in nest-building activities (Fig. 6B). This suggests that HPSE deficiency may protect against HSV-induced anxiety-like behavior due to reduced inflammation in the cortical and limbic neurons involved in stress responses. The marble burying test commonly used to assess anxiety and repetitive behavior showed that *Hpse+/+* mice buried significantly more marbles following HSV-1 infection compared to their mock-infected counterparts. This increase in marble-burying behavior is indicative of heightened anxiety and compulsive tendencies likely linked to HPSE-driven neuroinflammation and damage to dopaminergic and GABAergic neurons, which play a role in modulating anxiety responses. In contrast, *Hpse−/*− mice exhibited minimal changes in marble-burying behavior post-infection, suggesting reduced anxiety and lower compulsivity in the absence of HPSE (Fig. 6C). The ledge test, which assesses motor coordination and balance, revealed that HSV-1-infected *Hpse+/+* mice exhibited prolonged hesitation, frequent shaking, and balance difficulties when attempting to re-enter the cage. These impairments are consistent with neurodegenerative-like effects observed with HPSE-related inflammation likely impacting motor neurons and cerebellar structures. In contrast, *Hpse−/*− mice infected with HSV-1 showed no such deficits, performing similarly to uninfected controls (Fig. 6D), suggesting that HPSE may play a significant role in virus-induced motor coordination deficits. Finally, in the tape removal test, a measure of somatosensory function, both *Hpse+/+* and *Hpse−/*− mice showed increased latency in noticing and removing the tape after HSV-1 infection, with no significant differences between genotypes (Fig. 6E). This finding suggests that HPSE may not be a key factor in somatosensory deficits associated with persistent HSV-1 infection, potentially implicating viral damage to peripheral sensory neurons that are independent of HPSE-mediated pathways. Together, these findings indicate that HPSE-induced neuroinflammation and associated neuronal damage contribute detectably to neurobehavioral deficits, with *Hpse−/*− mice showing reduced anxiety, better memory, and improved motor coordination following chronic HSV-1 infection. This aligns with previous observations that HPSE amplifies neuroinflammatory and pyroptotic responses, underscoring the potential of HPSE inhibition to mitigate neurobehavioral impairments associated with viral infections in the central nervous system.

## DISCUSSION

While intranasal infection is relatively rare in humans, it directly disseminates HSV-1 to the brain, potentially leading to deadly or debilitating long-term consequences (33, 34). In this study, we provide direct evidence supporting a pathological role of HPSE in HSV-1 infection, building on previous findings that HSV-1 upregulates HPSE in a NF-κB-dependent manner during infection (21) and contributes to proinflammatory responses in the corneal epithelium (18). Long-term HSV-1 keratitis patients have elevated levels of HPSE in their tears (35). Here, using *Hpse−/*− mice, we extended the understanding of HPSE’s role in mediating brain inflammation, neurobehavioral changes, and disease outcomes, alongside investigating underlying molecular mechanisms.

HSV-1 encephalitis (HSE) is a severe CNS infection with limited therapeutic options; current antiviral treatments are not effective against the inflammatory components of HSE (36). Increasing evidence shows that uncontrolled neuroinflammation, rather than direct viral replication alone, is the primary driver of poor HSE outcomes (37, 38). In the context of viral brain infections, inflammation has emerged as a critical factor in injury, neurodegeneration, and behavioral abnormalities, a pattern seen in diseases like Japanese encephalitis, where neuroinflammation disrupts the blood–brain barrier (39). Our study highlights HPSE as a key regulator of neuroinflammation in HSV-1 infection, with HPSE-dependent inflammatory cascades involving multiple components, such as inflammasomes, caspases, gasdermin, and microglia activation, that exacerbate disease severity. In response to HSV-1 infection, pattern recognition receptors on brain cells, particularly microglia and astrocytes, detect viral components like proteins and viral RNA, triggering pathways that lead to NF-κB and IRF signaling. This results in transcriptional upregulation of immune response via chemokines and cytokines, such as *Ccl5*, *Il10*, and *Il6* (40). In our study, we observed exclusive microglial activation in the olfactory bulb (OL) of *Hpse+/+* mice, a significant finding as microglia is a primary source of NOS2 during neuroinflammation, producing cytotoxic nitric oxide that can contribute to neuronal damage (41). Our results suggest that in the presence of HPSE, TLR signaling is amplified, including the key pro-inflammatory cytokine, *Il17a*, which in turn promotes inflammasome activation, GSDMD cleavage, and pyroptotic cell death, further driving inflammatory damage to neuronal tissues.

Behavioral assays can be a valuable indicator of the brain regions impacted by HSV-1-induced neuronal injury and provide insights into the functions associated with those regions (42, 43). HPSE has previously been discussed with its possible involvement in aggressive behavior, but there are fewer reports in context of viral infections (26). Our data demonstrated that *Hpse−/*− mice outperformed *Hpse+/+* mice across several behavioral tests following chronic HSV-1 infection, suggesting a protective role of HPSE deficiency against virus-induced neurobehavioral impairment. For instance, the NOR test associated with memory and learning in the cortex and hippocampus revealed cognitive preservation in *Hpse−/*− mice, while *Hpse+/+* mice displayed significant deficits (Fig. 6A). The marble burying test, which reflects anxiety and compulsive behaviors linked to the cortex and amygdala, showed elevated repetitive behavior in infected *Hpse+/+* mice, indicative of increased anxiety potentially due to dopaminergic and GABAergic neuronal damage (44). In contrast, *Hpse−/*− mice displayed minimal anxiety-related changes, suggesting a mitigating role of HPSE deficiency on stress response pathways (Fig. 6B).

Furthermore, the ledge test, which assesses balance and coordination related to cerebellar and hippocampal function, revealed motor impairments in infected *Hpse+/+* mice, with longer re-entry times and balance issues consistent with neurodegeneration in motor-related regions (Fig. 6D). *Hpse−/*− mice, however, maintained a normal performance, indicating preserved motor coordination in the absence of HPSE. Our use of the tape removal test, measuring somatosensory function linked to the motor cortex and basal ganglia, did not show significant differences between genotypes post-infection, suggesting that somatosensory deficits may not be strongly HPSE-dependent (Fig. 6E).

Importantly, while the neurobehavioral changes observed in mice provide valuable insights, it is essential to recognize that these findings may not directly apply to humans. Humans possess maternal antibodies and have developed trained immunity due to HSV-1, which is a natural human pathogen. Additionally, circulating HSV-1 strains vary in neurovirulence, and humans are rarely subjected to intranasal HSV-1 infections. Nevertheless, the neurobehavioral impairments seen in *Hpse+/+* mice suggest that chronic HPSE-mediated neuroinflammation could contribute to cognitive and motor dysfunction over time. This aligns with human studies indicating a higher risk of neurodegenerative disorders, such as dementia, in individuals infected with HSV-1 (27).

**Fig 7:**
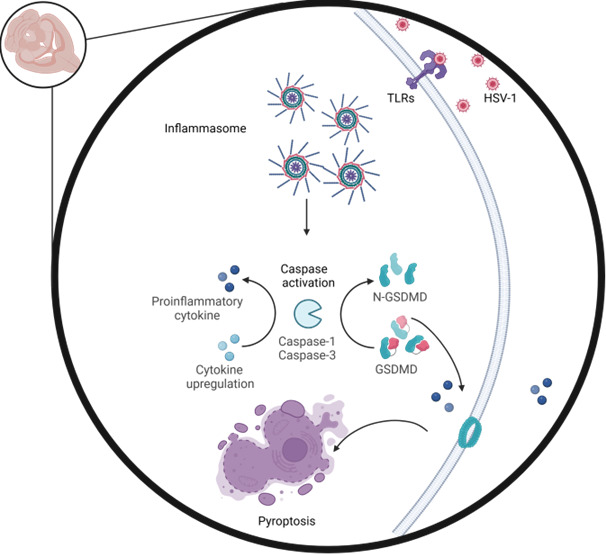
Overall schematic of HPSE-mediated neuroinflammation. After HSV-1 infection, HPSE was involved in cascade of events leading to inflammasome formation and caspase activation along with proinflammatory cytokine upregulation and gasdermin-mediated cell death. The above series of events leads to neuroinflammation and cell death.

In summary, this study underscores the pathological role of HPSE in promoting neuroinflammation and related neurobehavioral impairments following HSV-1 infection. In *Hpse+/+* mice, HPSE upregulation enhances inflammasome activation with an increase in proinflammatory cytokine production and leading to neuronal damage and behavioral deficits (Fig. 7). These findings suggest that targeting HPSE could be a promising therapeutic strategy for reducing HSV-1-induced neuroinflammation and preserving cognitive and motor function (28). Given the role of HPSE in HSV-1 spread, the observed long-term neuropathological effects could be a consequence of limited or absent virus replication in HPSE-deficient mice rather than HPSE actively inhibiting inflammasome activation. However, research in the cancer field, independent of viral infection, and our results more directly implicate HPSE as a potential trigger of inflammation. Future studies should aim to disentangle these possibilities by investigating the cause-and-effect relationship between HPSE and HSV-1, as well as the direct role of HPSE in driving neuroinflammation. Additionally, elucidating the mechanisms by which HPSE contributes to neuroinflammation in various neuronal cell types and assessing the potential of HPSE inhibitors in controlling both brain inflammation and disease severity in HSV-1 infections will be important objectives for future studies.

## MATERIALS AND METHODS

### Cells and viruses

Heparanase-positive (*Hpse+/+*) mouse embryonic fibroblasts (MEFs) and heparanase-deficient (*Hpse−/*−) MEFs were generously provided by Israel Vlodavsky (Technion—Israel Institute of Technology, Israel). MEFs used in this study were maintained at 37°C with 5% CO₂. HSV-1 (KOS-WT strain) was used for *in vitro* infections in MEFs, while the HSV-1 McKrae strain (provided by Dr. Patricia Spears, Northwestern University, USA) was used for *in vivo* infections. Viral stocks were propagated in Vero cells obtained from Dr. Spears and cultured in Dulbecco’s modified Eagle’s medium (DMEM; Corning) containing 10% FBS and 1% penicillin–streptomycin. Virus stocks were stored at –80°C.

### Mice

All mice were housed in the Biological Resources Laboratory (BRL) at the University of Illinois at Chicago under a standard light–dark cycle and provided with standard feed. For acute infection experiments, male and female 6- to 10-week-old C57BL/6J background (WT and *Hpse*-deficient) mice were anesthetized using the ketamine–xylazine method and intranasally infected with HSV-1 McKrae at 1 × 10⁷ PFU (Fig. S3). Mice were euthanized at 12 and 24 h post-infection (hpi) for qPCR analysis. Disease scoring was based on a standardized scale: 0: no symptoms, intact skin, and normal breathing; 1: mild nasal deskinning and mild swelling; 2: moderate nasal deskinning, minor ocular involvement, hydrocephalus, and mild breathing difficulty; 3: mild neurological symptoms, moderate swelling, and breathing difficulty; 4: extensive nasal and ocular deskinning and moderate neurological symptoms; and 5: severe breathing difficulty, severe neurological symptoms, and death. For chronic infection, mice received a lower dose (1 × 10^5^ PFU) and undergone behavioral tests 6 months post-infection. Mice were then euthanized, and brains were collected and preserved in formalin for immunohistochemistry.

### Quantitative polymerase chain reaction (qPCR)

Total RNA was extracted from mock- and HSV-infected *Hpse+/+* and *Hpse−/*− MEFs and from brain tissues of mock- and HSV-infected *Hpse+/+* and *Hpse−/*− mice using TRIzol reagent (Life Technologies). cDNA was synthesized using the High-Capacity cDNA Reverse Transcription Kit (Applied Biosystems), and qPCR was performed using Fast SYBR Green Master Mix (Applied Biosystems) on a QuantStudio 7 Flex System (Applied Biosystems). Primers were adopted as reported by Agelidis et al.(25). List of primers and reagents used for q-PCR analysis in this study are tabulated in S4 and S5.

### Hematoxylin and eosin (H&E) staining

Brain tissues embedded in OCT compound (Fisher HealthCare) were sectioned (10 µm) and stained with hematoxylin and eosin. Sections were dehydrated, cleared with xylene, mounted, and imaged on a Zeiss Axioskop 2 microscope.

### Immunohistochemistry

Brains were deparaffinized, rehydrated, and subjected to antigen retrieval in sodium citrate buffer (pH 6.0). Sections were permeabilized with 0.5% Triton-X, treated with 3% hydrogen peroxide, and blocked with a blocking buffer. After overnight incubation with primary antibodies at 4°C, sections were treated with HRP-conjugated secondary antibodies and developed with DAB. Slides were counterstained with hematoxylin, dehydrated, and mounted for imaging on a Zeiss LSM 710 confocal microscope.

### Western blot

Mock and HSV-1 infected *Hpse+/+* and *Hpse−/*− MEFs cells were used for western blot analysis. Cells were dissociated using a non-enzymatic cell dissociation solution and pelleted by centrifugation at 800 × *g* for 8 min. Cell pellets were resuspended in RIPA buffer (Sigma-Aldrich) with protease and phosphatase inhibitors (Thermo Fisher Scientific). Samples were incubated at 4°C for 30 min and centrifuged at 16,000 × *g* for 30 min. Supernatants were collected, and protein concentrations were determined and equalized using the BCA method. Each sample was mixed with LDS loading buffer (Life Technologies) and 5% β-mercaptoethanol (Thermo Fisher Scientific) and denatured at 90°C for 10 min. Proteins were separated by SDS-PAGE at 70 V for 3 h, transferred to nitrocellulose membranes (IB-23001, Invitrogen) using the iBlot two system, and blocked with 5% skim milk in TBST for 1 h at room temperature. Primary antibodies (diluted 1:1,000–1:5,000) were applied overnight at 4°C. Membranes were washed and incubated with HRP-conjugated secondary antibodies (1:1,000–1:5,000) for 1 h at room temperature. After final washes, blots were developed with SuperSignal West Pico or Femto substrate (Thermo Fisher Scientific) and visualized using the ImageQuant LAS4000 System (GE Healthcare Life Sciences).

### *Ex vivo* reactivation assay using plaque assay

Trigeminal ganglia were isolated from *Hpse+/+* and *Hpse−/*− mice after 24 hpi and cultured in DMEM. After 5 and 10 days, media from TG cultures were serially diluted and used to infect confluent Vero cells in 24-well plates. After 2 h, the inoculum was replaced with DMEM containing 0.5% methylcellulose. After three days of incubation, plaques were stained with crystal violet following methanol fixation, and PFU/mL was determined.

### Confocal microscopy

*Hpse+/+* and *Hpse−/*− MEFs were cultured in glass-bottom dishes (Cellvis LLC), infected with HSV-1 (KOS WT strain) at 5 MOI for 12 h, fixed with 4% paraformaldehyde and permeabilized with 0.1% Triton-X (Thermo Fisher Scientific) for 15 min at room temperature. Cells were stained with primary antibodies for 1 h, followed by Alexa Fluor-conjugated secondary antibodies and NucBlue for 1 h at room temperature. Imaging was performed on a Zeiss LSM 710 confocal microscope.

### Gasdermin D activation after HSV-1 infection

*Hpse+/+* and *Hpse−/*− MEFs were infected with HSV-1 (KOS WT strain) at an MOI of 0.1 for 2 h, after which DMEM was added. Cells were harvested at 12 and 24 hpi, and Gasdermin D activation was analyzed by western blot.

### Behavioral assays

#### Novel object recognition (NOR) test

The NOR test assesses memory by measuring preference for a novel object. Male mice were habituated in a polypropylene box and then exposed to two identical objects. On the third day, one object was replaced with a novel one, and exploration time was recorded (32).

#### Marble burying test

Male mice were placed in a cage with bedding and 10 marbles arranged in two lines. After 30 min, buried marbles were counted, assessing anxiety and repetitive behavior (44).

#### Nestlet shredding test

Male mice were given a weighed nestlet in a cage for 6 h, and shredded nestlet percentage was calculated as a measure of stress and nesting behavior (44).

#### Tape removal test

Sensorimotor function was evaluated by applying adhesive tape to a hind paw and measuring the time taken by male mice to notice and remove the tape (45).

#### Ledge test

Coordination was assessed by placing male mice on a ledge and observing their balance and movement back into the cage (46).

### Statistical analysis

GraphPad Prism software was used to analyze the data and create the graphs. One way analysis of variance (ANOVA), two-way ANOVA with multiple comparison, and nonparametric *t*-tests were used wherever applicable to determine significant differences as denoted in figure legends.

## SUPPLEMENTAL MATERIAL

Supplemental material mbio.03765-24-s0001.pdf Figures S1 to S5.

ASM does not own the copyrights to Supplemental Material that may be linked to, or accessed through, an article. The authors have granted ASM a non-exclusive, world-wide license to publish the Supplemental Material files. Please contact the corresponding author directly for reuse.
