# Supplementary material for: HPSE-mediated proinflammatory signaling contributes to neurobehavioral deficits following intranasal HSV-1 infection
Source: mBio. 2025 Feb 27;16(4):e03765-24. doi: 10.1128/mbio.03765-24 (PMC11980599; doi:10.1128/mbio.03765-24)
Supplement: Supplemental material — Figures S1 to S5. [file mbio.03765-24-s0001.pdf]

HPSE-Mediated Proinflammatory Signaling Contributes to Neurobehavioral Deficits Following Intranasal HSV-1 Infection

Hemant Borase[1], Chandrashekhar D Patil[1], Tibor Valyi-Nagy[2], and Deepak Shukla[1,3*]

[1] Department of Ophthalmology and Visual Sciences, [2] Department of Pathology, Neuropathology Service, [3] Department of Microbiology and Immunology, University of Illinois Chicago, Chicago, IL 60612

*Corresponding author dshukla@uic.edu

Supplementary figure legends:

Fig. S1-A- *Ex-vivo* HSV-1 reactivation after 5- and 10-days post addition of TG into growth media and B- Microglial count in OL of *Hpse+/+* and *Hpse -/-* mock and HSV-1 infected mice.

Fig. S2 Schematics of different behavioral assays performed to assess behavioral impact of HSV-1 infection in WT and *HPSE -/-*. (A)- Novel Object Recognition (NOR) test. (B) Marble Burying test. (C) Nestlet Shredding test. (D) Ledge test and (E) Tape Removal test.

Fig. S3 A representative Schematic of *in vivo* experiment performed on *Hpse+/+* and *Hpse -/-* mice. The mice were infected with 1 X10^7 PFU of HSV-1 (Acute infection) and euthanized after 12 and 24 hpi. For behavioral assays mice were infected with 1 X10^5 PFU of HSV 1 and behavioral assays were done after 6 months post infection.

S4- List of primers used for q-PCR analysis in this study.

S5- List of key reagents used in this study.

Supplementary file 1

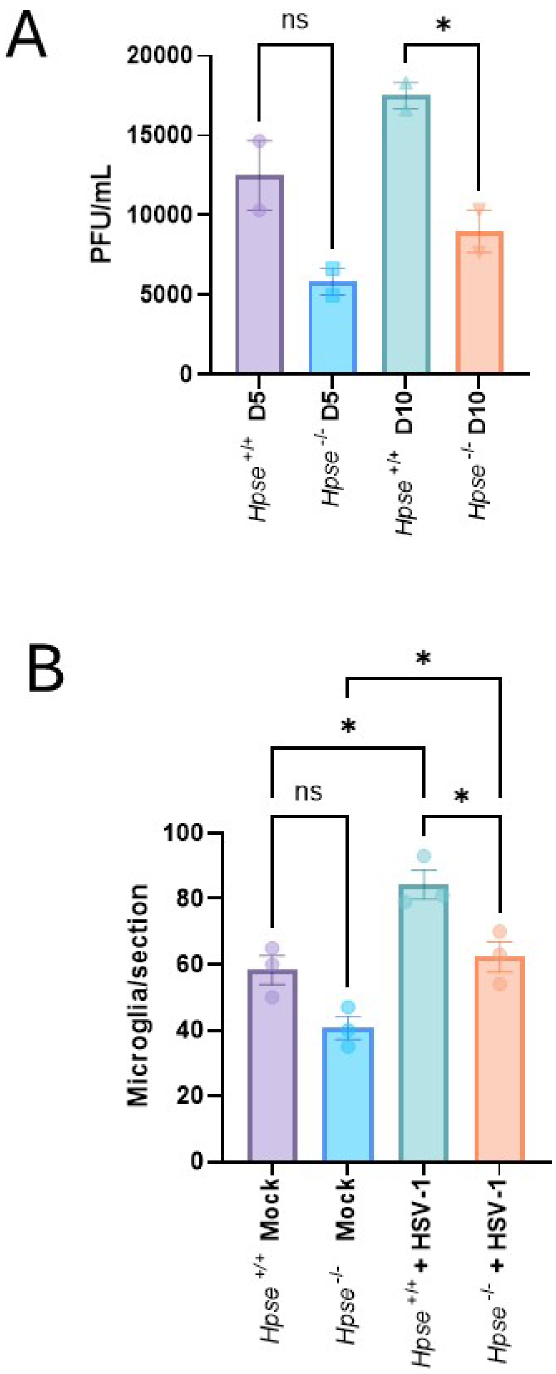

Supplementary file 2

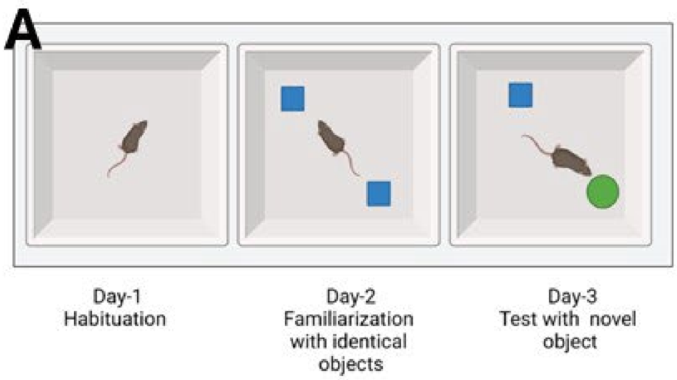

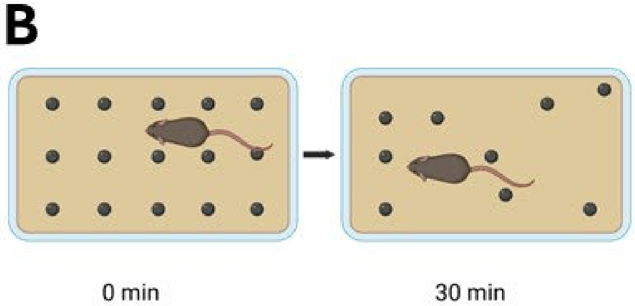

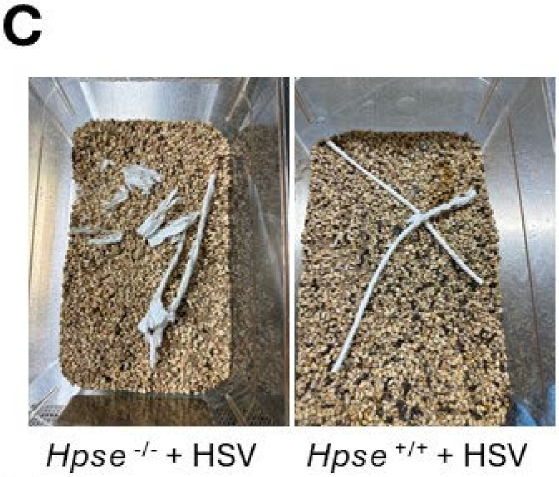

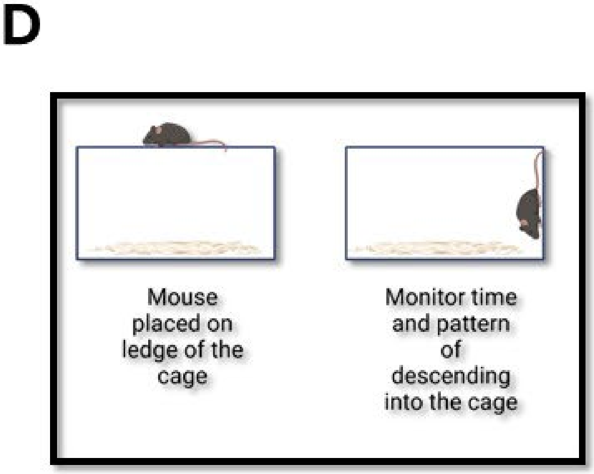

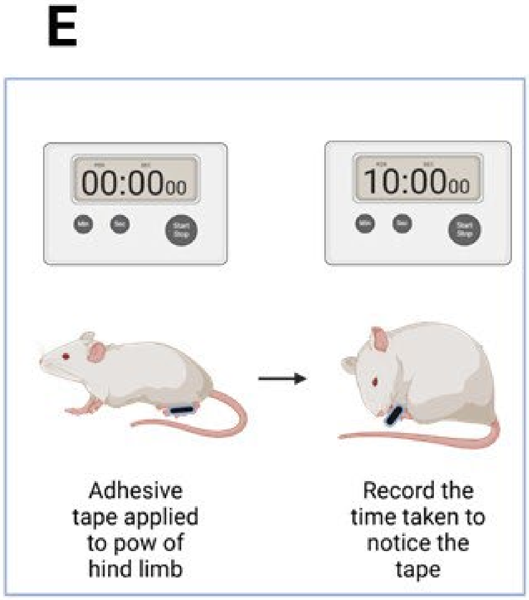

Supplementary file-3

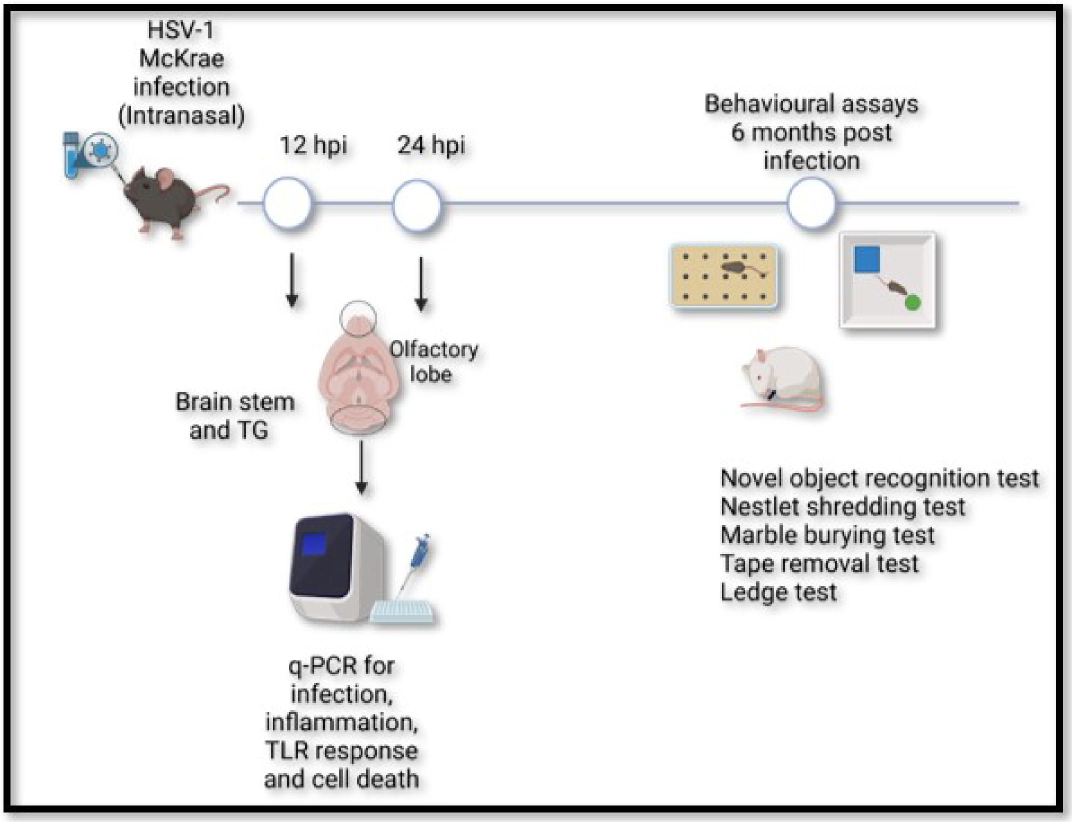

Supplementary file-4

Gene	Forward (5'-3')	Reverse (5'-3')
Actb	GGCTGTATTCCCCTCCATCG	CCAGTTGGTAACAATGCCATGT
Gapdh	TTCACCACCTAGGAGAAGG	AGAAGGGGCGGAGATGAT
Il17a	GTCAATGCGGAGGGAAAG	CACGAAGCAGTTTGGGAC
Ccl5	ATATGGCTCGGACACCACTC	GATGCCGATTTTCCCAGGAC
Il10	TAATAAGCTCCAAGACCAAG	TAGAATGGGAACTGAGGTATC
Nos2	CAGCACAAGGGGTTTTCTTC	AACGGAGAACGTTGGATTTG
Tlr3	GTGAGATACAACGTAGCTGACTG	TCCTGCATCCAAGATAGCAAGT
Tlr4	ATGGCATGGCTTACACCACC	GAGGCCAATTTTGTCTCCACA
Tlr7	GTTCTATGGAGAGCCGGTGATA	ATTCTTTAGATTTGGCGGCATA
Tlr9	ATGGTTCTCCGTCGAAGGACT	GAGGCTTCAGCTCACAGGG
Casp1	CCGAGGGTTGGAGCTCAAG	TTCACCATCTCCAGAGCTGTGA
Casp3	TGGCATTGAGACAGACAGTGG	CCAGGAATAGTAACCAGGTGCTG
Nlrp3	CTGAACCTGGGCAACAATGA	ACATTTCACCCAACTGTAGGCTC
gB	GGACATCAAGGCGGAGAACA	TTCTCCTTGAAGACCACCGC

Supplementary file-5

REAGENT or RESOURCES	SOURCE	IDENTIFIER
**ANTIBODIES (Dilution)**		
**GAPDH (1:5000)**	Proteintech	10494-1-AP
**gB (1:5000)**	Santa Cruz Biotechnology	10B7
**Gasdermin D (1:1000) for WB**	Abclonal	A17308
**Gasdermin D (1:100) for Confocal**	Abclonal	A17308
**HPSE (1:2000)**	Proteintech	66226-1-Ig
**Iba-1 (1:100)**	abcam	ab178846
**p-NFKB (1:100)**	Proteintech	82335-1-RR
**Anti-rabbit IgG HRP- linked antibody (1:3000)**	Cell Signaling Technology	7074S
**Anti-mouse IgG HRP-linked antibody (1:3000)**	Cell Signaling Technology	7076S
**DMEM basic (1X)**	Corning	10-013CV
**Opti-MEM (1X)**	Gibco	31985-070
**Trypsin**	Corning	25-052-CV
**Penicillin-Streptomycin**	Corning	30-002-CL
**Fetal bovine serum (FBS)**	Sigma-Aldrich	22g086-A
**Trizol**	Thermo Scientific	15596018
**c-DNA kit**	Applied Biosystems	4368814
**qPCR master mix**	Applied Biosystems	4385612
**IHC Kit**<br>**Mouse and Rabbit Specific HRP/DAB IHC Detection Kit - Micro-polymer**	abcam	Ab236466
**Hematoxylin**	Thermo Scientific	6765003
**Eosin**	Sigma-Aldrich	HT110316-500ml
